# Improving access to chronic pain care with central referral and triage: The 6-year findings from a single-entry model

**DOI:** 10.1080/24740527.2023.2297561

**Published:** 2024-01-12

**Authors:** Tania Di Renna, Emeralda Burke, Anuj Bhatia, Hance Clarke, David Flamer, John Flannery, Andrea Furlan, Dinesh Kumbhare, James Khan, Karim Ladha, Howard Meng, Andrew Smith, David Sussman, Rachael Bosma

**Affiliations:** a Toronto Academic Pain Medicine Institute, Women’s College Hospital, Toronto, Ontario, Canada; b Department of Anesthesiology, University of Toronto, Toronto Western Hospital, Toronto, Ontario, Canada; c Department of Anesthesia and Pain Medicine, Toronto General Hospital, Toronto, Ontario, Canada; d Department of Anesthesiology, University of Toronto, Sinai Health, Toronto, Ontario, Canada; e Musculoskeletal Rehabilitation Program, Toronto Rehabilitation Institute, Toronto, Ontario, Canada; f Department of Anesthesia, St. Michael’s Hospital, Toronto, Ontario, Canada; g Department of Anesthesiology and Pain Medicine, Sunnybrook Health Sciences Centre, Toronto, Ontario, Canada; h Department of Psychiatry, Centre for Addiction and Mental Health, University of Toronto, Toronto, Ontario, Canada

**Keywords:** Single-site entry, intake, triage, chronic pain

## Abstract

**Background:**

Despite the established efficacy of multidisciplinary chronic pain care, barriers such as inflated referral wait times and uncoordinated care further hinder patient health care access.

**Aims:**

Here we describe the evolution of a single-entry model (SEM) for coordinating access to chronic pain care across seven hospitals in Toronto and explore the impact on patient care 6 years after implementation.

**Methods:**

In 2017, an innovative SEM was implemented for chronic pain referrals in Toronto and surrounding areas. Referrals are received centrally, triaged by a clinical team, and assigned an appointment according to the level of urgency and the most appropriate care setting/provider. To evaluate the impact of the SEM, a retrospective analysis was undertaken to determine referral patterns, patient characteristics, and referral wait times over the past 6 years.

**Results:**

Implementation of an SEM streamlined the number of steps in the referral process and led to a standardized referral form with common inclusion and exclusion criteria across sites. Over the 6-year period, referrals increased by 93% and the number of unique providers increased by 91%. Chronic pain service wait times were reduced from 299 (±158) days to 176 (±103) days. However, certain pain diagnoses such as chronic pelvic pain and fibromyalgia far exceed the average.

**Conclusions:**

The results indicate that the SEM helped reduce wait times for pain conditions and standardized the referral pathway. Continued data capture efforts can help identify gaps in care to enable further health care refinement and improvement.

## RÉSUMÉ

**Contexte:** Malgré l’efficacité établie des soins multidisciplinaires dans le traitement de la douleur chronique, des obstacles tels que des délais d’attente prolongés et l’absence de coordination des soins entravent davantage l’accès des patients aux services de santé.

**Objectifs:** Nous décrivons ici l’évolution d’un modèle à entrée unique visant à coordonner l’accès aux soins pour la douleur chronique dans sept hôpitaux de Toronto. Nous examinons également l’effet de ce modèle sur les soins aux patients six ans après sa mise en œuvre.

**Méthodes:** En 2017, un modèle à entrée unique novateur a été mis en place pour orienter les patients souffrant de douleur chronique à Toronto et dans les régions avoisinantes. Les patients sont reçus de manière centralisée, triés par une équipe clinique et un rendez-vous leur est attribué en fonction du degré d’urgence et de l’établissement de soins ou du prestataire le plus approprié.

Pour évaluer l’impact du modèle à entrée unique, une analyse rétrospective a été entreprise afin de déterminer les schémas de consultation, les caractéristiques des patients et les temps d’attente pour les demandes de consultation au cours des six dernières années.

**Résultats:** La mise en œuvre d’un modèle à entrée unique a permis de rationaliser le nombre d’étapes du processus de demande de consultation et a conduit à l’élaboration d’un formulaire de demande de consultation normalisé comprenant des critères d’inclusion et d’exclusion communs à tous les sites. Au cours de la période de six ans, le nombre de demandes de consultation a augmenté et le nombre de prestataires uniques a augmenté de 91 %.

Les temps d’attente pour les services de traitement de la douleur chronique ont diminué de 299 (±158) jours à 176 (±103) jours. Cependant, certains diagnostics de douleur, comme la douleur pelvienne chronique et la fibromyalgie, dépassent de loin la moyenne.

**Conclusions:** Les résultats indiquent que le modèle à entrée unique a contribué à réduire les temps d’attente pour les affections douloureuses et à normaliser le parcours de consultation. La poursuite des efforts de collecte des données peut aider à recenser les lacunes dans les soins, permettant ainsi une amélioration continue des soins de santé.

## Introduction

Chronic pain, defined as pain that lasts longer than 3 months, affects about one in five Canadians.^1^ It interferes with quality of life as well as social and family relationships.^2^ Given this multidimensional nature, the management of chronic pain focuses on the biopsychosocial model.^3^ Effective use of a biopsychosocial model involves interdisciplinary care and integrates medical (pharmacological and procedural), physical, psychological, as well as self-management strategies.^3^

Despite clinical guidelines promoting interprofessional models of care that address the biopsychosocial factors influencing pain, chronic pain management in Canada is complex and continues to be fragmented.^4^ There are significant challenges in the system that impede access to care, including lack of public funding and reimbursement, distance from urban centers, substantial wait times in many jurisdictions, lack of coordination across health care providers, and language and cultural barriers.^1,5,6^ Pain management specialists are often distributed across hospitals, with each clinic providing specialized services, making it challenging for referring providers to identify the most appropriate center for their patient to receive care. A lack of coordinated services leaves patients vulnerable to multiple referrals, long wait times, and unimodal care, often leading to frustration and poor health outcomes.^5,7^

The single-entry models (SEM) is a promising strategy to improve wait times in health care, and these models have been successfully implemented for bariatric and arthroplasty operations.^8^ In addition, this model can standardize the patient journey to provide more efficient system navigation and ensure that the patient is obtaining equitable and timely access to care.^7^ SEMs typically consolidate patient referrals for a group of specialists into a single wait list. This can be done by having a single point of entry for all referrals (centralized intake).^9^ Patients are then prioritized by urgency (centralized triage) and presenting symptoms or diagnosis. They are then assigned to the next available and the most appropriate specialist.^9^ Appropriate assignment to health care providers who are well equipped to support the specific care needs of patients is critical. SEMs require an iterative process of quality improvement where feedback is provided by patients, referring health care providers, and those who treat these patients, and the centralized collection of patient and provider data helps to identify gaps in care.^10^ The quasi-objective criteria of urgency and necessity in triaging patients within SEMs helps improve equity and access to treatment.^10^ Previous research has highlighted patient and provider satisfaction after SEMs were implemented.^11,12^

Single-entry systems are most common in contexts with well-defined patient populations and standardized procedures.^13^ However, few SEM studies have been operationalized in the chronic pain setting.^11,14^ Chronic pain encompasses a wide array of conditions and clinical subspecialties and requires multidisciplinary and interdisciplinary treatment strategies that are tailored to the individual patient’s needs and preferences.^1^ It is unclear from the existing literature how a centralized referral and triage system will function in a chronic pain setting where there is variability in the reason for referral and in the clinical subspecialty and services available at clinical sites.

Wait time and health system navigation challenges, as well as a limited fixed budget for care delivery, led to the development of the Toronto Academic Pain Medicine Institute (TAPMI) to serve patients with chronic pain in Toronto and the surrounding areas. TAPMI is a hub-and-spoke SEM. The “hub” site, housed in Women’s College Hospital, manages centralized intake and triage. Referrals are then distributed to five “spoke” sites (academic hospitals) to provide specialized services: Women’s College Hospital, Sinai Health System, St. Michael’s Hospital, University Health Network, and Center for Addiction and Mental Health. TAPMI receives referrals from across the Greater Toronto Area, which covers a population of approximately 6 million people. Here we describe the evolution of TAPMI’s SEM and describe its long-term impact at 6 years after implementation. Understanding this potential is important, because SEMs may enhance health system navigation and support timely access to interdisciplinary chronic pain care.

## Materials and Methods

### Pre-Audit Single-Entry Model

In 2016, prior to the implementation of TAPMI’s centralized referral process, we conducted an environmental scan to detail the current state of clinical processes from referral to service delivery. We solicited feedback from 25 patients, physicians, and community care providers. We determined the number of steps in the referral pathway and examined the referral form requirements associated with referrals sent from community care settings to each tertiary clinic in the partnership. We also collected extensive service inventories from each partner site to assign patients to the most appropriate clinic and services.

### Development of a Single-Site Entry Model

In partnership across the five academic institutions that comprise the Toronto Academic Pain Medicine Institute, we co-developed an SEM beginning in 2017. This system included establishing a centralized intake process where all referrals for patients requiring tertiary-level pain care were sent to the centralized location via fax. We created a standardized referral form through an iterative and collaborative process that included three quality improvement cycles (plan, do, study, act) in which we analyzed the completion of 80 referral forms and made improvements based on feedback from partner sites and advisors from primary care settings. This form was created to alleviate the burden on primary care practitioners of filling out lengthy referral forms while simultaneously providing enough information for the triage team to send the patient to the appropriate site/chronic pain care provider.

All referrals are received at a centralized location where they are processed and undergo a rigorous clinical review and triage process. Though the professional complement of the triage team has changed over time, currently the triage team comprises three registered practical nurses. A senior pain medicine physician acts as a supervisor and an expert reviewer and discusses any concerns identified by the triage nurses.

**Figure 1.**
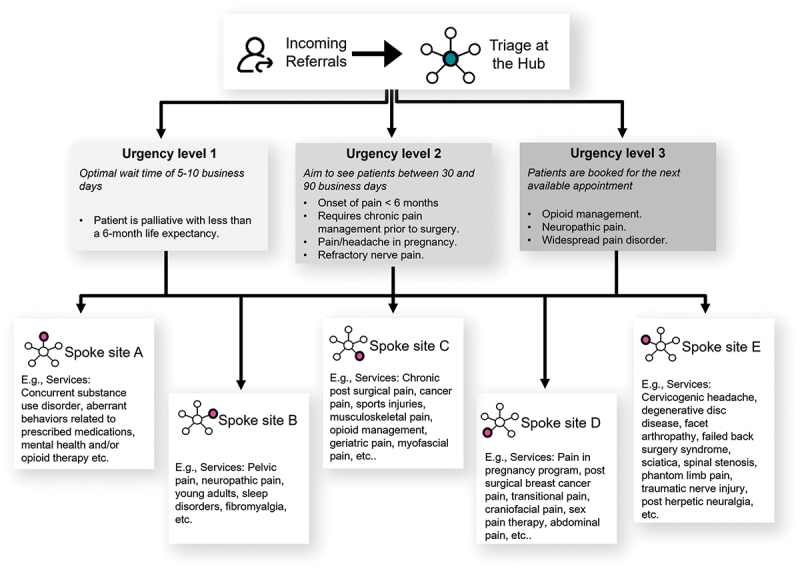
An overview of our triage process and examples of the services offered at each spoke site.

Each referral is reviewed by a member of the triage team to ensure that they are appropriate for TAPMI services and for completion. If the form is incomplete, the triage team requests any missing documentation from the referring health care provider (physician or nurse practitioner). All accepted referrals are triaged to the most appropriate clinical team across the partnership according to the urgency (level 1: 5–10 workdays, level 2: 10–30 business days, level 3: next available appointment), the patient’s pain condition, services requested, and capacity of the clinic (Figure 1). The referral package is then forwarded to the assigned site, the referring provider is notified, and the patient is scheduled by the assigned site for services at that location.

### Post-Implementation Phase of Central Triage

Six years have passed since this centralized system was implemented. The impact of this centralized system was described retrospectively over a 6-year period, occurring from April 1, 2017, to March 31, 2023. This initiative was formally reviewed by institutional authorities at Women’s College Hospital and was deemed not to require research ethics board approval. The extracted data characterized patterns in referrals and patient characteristics and included the following: the number of referrals and unique referring providers for each year, the top five referring providers by specialty, and the proportion of urgency levels over the 6 years. The top reasons for pain diagnosis divided into categories and specific conditions over the 6 years are described, as well as how these trends have changed annually. Our patient demographic characteristics including sex and age are identified. The geographical proportion of our referrals over the 6 years and the proportion of interpreters requested including the total number of languages and the top five languages are also listed. Lastly, we analyzed the proportion of our patient population who identify as marginalized using the Ontario Marginalization Index (ON-Marg). The ON-Marg is a data tool that combines a range of demographic indictors into four distinct dimensions of marginalization.^15^ The four dimensions are (1) residential instability, (2) material deprivation, (3) dependency, and (4) ethnic concentration.^15^ Residential instability measures area-level concentrations of people experiencing high rates of family or housing instability. Material deprivation refers to individuals’ and communities’ access to basic material needs using indicators including income, quality of housing, educational attainment, and family structure. Dependency represents area-level concentrations of people who do not have income from employment. Ethnic concentration relates to high area-level concentrations of people who are recent immigrants and/or people belonging to a “visible minority” group. All results are reported through descriptive statistics.

Examining the impact of implementing an SEM within the context of chronic pain, we also describe the mean wait times and standard deviations for clinical appointments over time and highlight deviations in the mean wait times for specific patient populations.

## Results

### Pre-Audit Phase

Results from the pre-audit environmental scan revealed redundancies in the referral pathway in which referring providers often submitted referrals to several pain clinics for the same patient, causing artificially inflated waitlists. Furthermore, each clinical site had its own referral form, inclusion/exclusion criteria, and triage process, with an average of five steps from receipt of referral to the scheduling of appointments. There were different service offerings in each clinic that were not openly advertised and an overall lack of harmony in care among the different sites. Recommendations that arose from the environmental scan included the development of a single-site entry system in which there was one common referral form, one point of contact for services, coordinated care across sites, and a clear process that increases the likelihood of a referred patient being treated promptly.

With the creation of our SEM, we now have a standardized referral form with common inclusion and exclusion criteria. Clinics continue to be subspecialized and triage is based on eligibility for the patients across the extensive services provided at each clinic. Referrals triaged to partner sites now require only a brief administrative review to support appointment scheduling, thereby substantially reducing the burden of triage review at each partner site.

**Figure 2.**
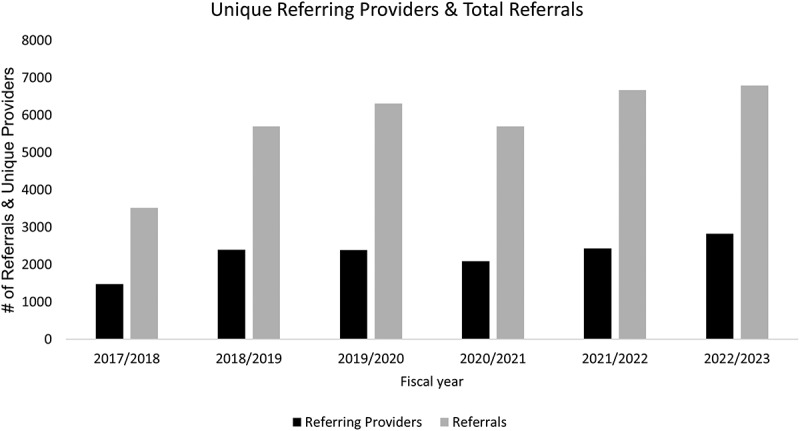
Number of referring providers and referrals per fiscal year.

Over 6 years of implementation of a centralized referral and triage process, there was an 93% increase in the number of referrals from 3520 in 2017–2018 to 6796 in 2022–2023. This parallels the increase in the number of unique referring providers (91%): there were 1480 unique referring providers in 2017–2018 and 2825 in 2022–2023 (Figure 2).

Overall, the leading specialties of referring providers include family and community medicine, community anesthesiology and pain medicine, general internal medicine, physical medicine and rehabilitation, and nurse practitioner.

**Figure 3.**
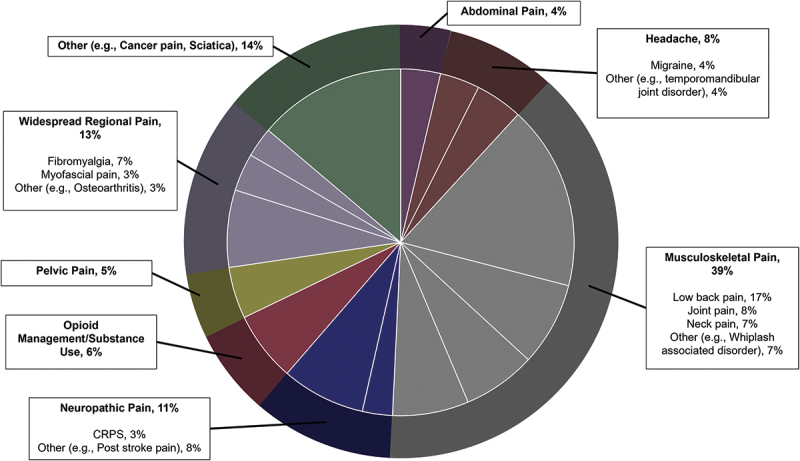
Top five referral diagnoses over 6 years (2017–2018 to 2022–2023). CRPS = complex regional pain syndrome.

The majority (88.49%) of referred patients were referred for the next available appointment (not deemed urgent), 11.22% were assigned a medium level of urgency with recommendations to be seen within 10 to 30 working days, and 0.28% were urgent and advised to be scheduled for an appointment within 5 to 10 working days. Across 6 years, the reason for referral across broad pain categories included musculoskeletal pain, widespread pain disorders, neuropathic pain, headache, opioid management/substance use issues, pelvic pain, abdominal pain and, other pain (Figure 3). Of all pain conditions, low back pain was the most referred pain diagnosis in each of the years, with neck pain and limb joint pain ranking second and third, respectively, for three of the years. In the first year of centralized intake, chronic pelvic pain was a common reason for referral, and fibromyalgia and headaches became more prevalent reasons for referral over time. Other common reasons for referral included cancer pain, rheumatological conditions, and traumatic brain injury.

**Figure 4.**
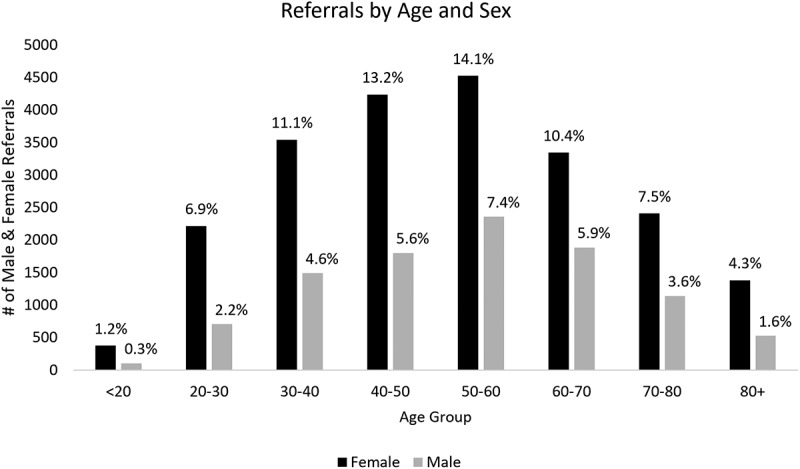
Number of referrals by age and sex over 6 years (2017–2018 to 2022–2023).

Thirty-one percent of referrals were for males and 69% were for females. A large proportion of patients were between 40 and 60 years of age (40%; Figure 4).

The catchment area of our referrals included Toronto (61%), the Greater Toronto Area (22%), and other areas in Ontario (17%).

Using the ON-Marg Index, 22% of our patient population identified as marginalized. This has remained relatively consistent over the 6 years, remaining between 20% to 22%. The ON-Marg Index uses four indicators to understand the marginalization of a specific geographical area. Residential instability across the 6 years was 58%, with a range between 55% and 60% each year. There was an increase in ethnic concentration from 2017–2018 (59%) to 2022–2023 (62%). Material deprivation across the 6 years was 36%, with a range between 34% and 36%. Lastly, the proportion of the patient population that was marginalized according to dependency was 28% across the 6 years (range = 26%–29%). Over the 6 years, 1% of our patient population requested an interpreter, for a total of 35 languages. The top five languages for which interpreters were requested were Portuguese, Spanish, Cantonese, Russian, and Farsi.

**Figure 5.**
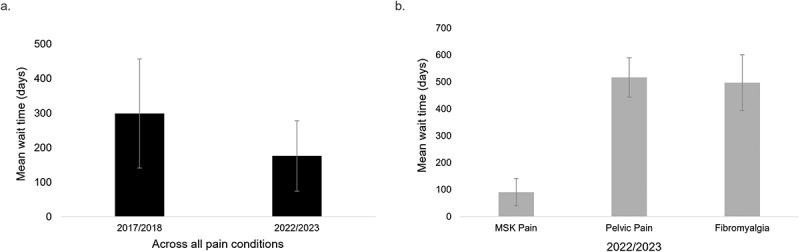
(a) The mean wait time (in days) for chronic pain services for urgency level 2 and 3 patients. (b) The mean wait time in 2022–2023 for patients with MSK-related pain, pelvic pain, and fibromyalgia.

The wait time for urgency level 1 referrals has been maintained at less than 10 working days. The mean wait time for chronic pain services for urgency level 2 and 3 patients decreased from 299 (±158) days in the first year to 176 (±103) days in the sixth year (*t* = 4.59, *P* < 0.0001; Figure 5a). However, there was substantial variation in the wait times depending on the subspecialty services for pain disorders. For example, in 2022–2023 the wait time for fibromyalgia pain was a mean of 517 (±73) days and, for chronic pelvic pain, it was a mean of 496 (±103) days, whereas for musculoskeletal pain (low back, neck pain) the mean wait time was 91 (±50) days (Figure 5b).

## Discussion

The TAPMI SEM is an innovative model for chronic pain care that brings together a centralized referral and triage process to enhance transparency, support health system navigation, and facilitate timely access to care. With the creation of our SEM system, we now have a common referral form and one point of contact for referral management. Centralized triage based on eligibility for the patients across the extensive services provided at each clinic enables a clear process for referring providers that increases the likelihood of successful referrals. Patients are seen by physicians capable of treating their condition sooner with a central triage model. Over the past 6 years, we have substantially improved the average wait times for chronic pain services for urgency level 2 and 3 patients and prioritized care based on urgency, with urgent (level 1) patients seen within 5 to 10 business days. Our single-site entry system has also enabled us to track referral volume and characteristics over time to respond rapidly to changes in the system and identify gaps in services.

Over a 6-year period, there has been a substantial increase in the number of referrals and referring providers. Upon development of the SEM, we implemented several strategies to disseminate and raise awareness regarding our services. This included various presentations with family health teams and networks, sharing through social media and e-mail communication channels, and the development and promotion of our website, which highlights our program and enables access to resources, including the common referral form. Primary care providers report that over one-third of appointments with adults in a typical week involve patients with chronic pain and that it is complex and challenging to manage.^16^ Combined with an ~6% increase^17^ in the population of our catchment area over this time period, it is possible that an enhanced awareness of the TAPMI chronic pain service model and demands for increased pain services contributed to the increase in referral volumes received.

The characterization of the information provided through central triage has underscored identified gaps in care and shaped clinical program development. Using the information acquired through this single-entry system, we were able to allocate clinical programming resources according to patient referral patterns in the reason for referrals. For example, approximately 39% of referrals were for patients with musculoskeletal (MSK) pain. MSK conditions are leading contributors to disability worldwide and, with population growth and aging, the number of people living with MSK pain is rapidly increasing.^18,19^ In response, interprofessional clinical services for MSK-related pain were developed across multiple TAPMI partner sites to address referral volumes and reduce wait times.

Through central triage, we were also able to identify gaps in clinical services in which specific chronic pain conditions were underserved or, in some cases, for which there were no services available. For example, between 2017 and 2019 there were no services available across the TAPMI partnership for patients with chronic pelvic pain. Because chronic pelvic pain is a common, debilitating condition that affects approximately 26% of the global female population^20^ and as many as 1 in 10 people assigned male at birth,^21^ we recognized that this represented a large segment of the chronic pain population that had little to no options in terms of programs to be referred to for their chronic pelvic pain in our catchment area. During this period, chronic pelvic pain was one of the top reasons for referral, demonstrating a significant need for care options for this population. In response to this need, we developed an interprofessional chronic pelvic pain program that includes services from pain medicine, gynecology, psychology, and pelvic physiotherapy. We also identified gaps in health care services for patients with fibromyalgia, young adult care (patients transitioning from pediatric care services), and transgendered pelvic pain care, as well as much needed interventional cancer pain care. In response, we have developed new programs to serve these populations.

Monitoring the demographic and patient characteristics of the referrals that we receive has also informed how we approach matters of equity, diversity, and inclusion; for example, being intentional in considering the social conditions that our patients live in or may be exposed to when designing programming and access to care (e.g., virtual versus in-person, group-based versus individual care, the need for interpretation services). This also includes tailoring our programs to meet social needs; for example, recognizing that many patients are between ages 40 to 60 years and thus developing programming that focuses on return to work and functional work-related goals. This also included hiring more female practitioners because the data informed us that 69% of patients were female while 90% of practitioners were male. Capturing information regarding referring providers also enabled us to track individuals who referred a high volume of patients and used e-consult through our mobile clinic program to support community care.

Many outpatient clinicians use a “multiple-queue, multiple-server” model in which each specialist has a separate queue; if one specialist’s schedule is full, a new patient who is referred to them will have to wait even if another specialist is available.^9,11^ Different referral processes and wait list management strategies further exacerbate system inefficiencies and can lead to inequitable access to care.^9,11^ Consistent with previous findings, implementing a single-entry system for chronic pain facilitated efficiency by reducing wait times.^8,11,12^ Though single-site entry models for other clinical services (e.g., surgery) or for unimodal specialist care have been demonstrated to promote equity by reducing the variability of wait times between specialists,^8,14^ specialists in pain medicine have diverse professional backgrounds, comprise interprofessional care teams, and serve a diverse patient population with many chronic pain conditions. In our model, triage for chronic pain services requires not only common inclusion and exclusion criteria for the program but a working and timely knowledge of the inventory of services and the distinctions between pain specialty sites, eligibility criteria for each service, as well as the wait times across services to enable easier access to the right care. The current model also allows patient triage to subspecialists and for pooling of scarce allied health resources such as mental health counseling, physical therapy, and pharmacy at one location and allows convenient referral.

Although wait times more than 6 months have been deemed medically unacceptable,^22^ wait times for interprofessional chronic pain care can range from 6 months to 2½ years, and more than 50% of patients are not seen within the recommended wait time.^6,22^ Implementing a single-site entry model for chronic pain led to an overall reduction in the average wait time for patients with chronic pain. However, for many patients, the wait times remain longer than recommended. For example, wait times remain more than two and a half times the recommended time for patients seeking services for chronic pelvic pain or fibromyalgia. This creates inequities in the access to care and deterioration in patients’ health, functioning, and quality of life.^1,3^ Furthermore, evidence suggests that waiting for interprofessional care negatively impacts treatment outcomes once patients do receive treatment from an interprofessional pain clinic.^23^ Single-site entry systems provide opportunity to closely monitor referral volume and service needs and create system efficiencies that support health system navigation and reduce wait times. However, the increasing burden of disease and scarcity of clinical capacity necessitate that additional innovative strategies be employed.^14,24^

Our evaluation was limited by the retrospective study design, which made it challenging to extract and compare high-quality data across all metrics of interest. Findings from other SEMs have highlighted the need to design centralized referral and triage processes with the data capture infrastructure, which enables referrals to be systematically documented and real-time wait time data to be shared across partner sites.^14^ Our SEM was implemented without the adoption of a centralized wait list database or a referral management system. Because our partner sites have different electronic medical records systems, communication regarding triage occurred through printed fax and updates to wait time information and service inventory were communicated through e-mail correspondence, making it difficult to support the systematic distribution of referrals across sites. Opportunities to enhance the data capture through a central referral process include acquisition of information regarding patient preference and accessibility to inform triage decisions and support the creation of personalized care pathways.

Our single-site entry system was created through collaboration between the clinical and administrative leads across all partner sites to reduce the burden of health system navigation for referring providers and patients and improve access to chronic pain care. The success and findings from central data capture highlight the opportunity to identify gaps in service and system inequities and has also enabled us to quantify the clinical capacity required to meet increasing demand for pain services as our population ages.
